# Chylous ascites as the main manifestation of left ventricular dysfunction: a case report

**DOI:** 10.1186/1471-230X-5-25

**Published:** 2005-08-03

**Authors:** Ezequiel Ridruejo, Oscar G Mandó

**Affiliations:** 1 Hepatology Section, Department of Internal Medicine, Centro de Educación Médica e Investigaciones Clínicas "Norberto Quirno" (CEMIC), Las Heras 2939, (1425), Buenos Aires, Argentina

## Abstract

**Background:**

Ascites is one of the most common complications of liver diseases, even though in 15% of the cases it is related to extrahepatic diseases; 3% are of cardiac nature and they appear associated with signs and symptoms of heart failure.

**Case presentation:**

A 70 year old man was admitted with more than one year history of abdominal distension and a weight gain of 10 kilograms. He is asymptomatic and walks 2000–3000 meters a day without angor or dyspnea. The physical examination shows moderate abdominal distension, with no hepatosplenomegaly or edema, and there is mild jugular vein distension. The studies performed (complete laboratory work up, paracentesis, liver biopsy, echocardiogram, intrahepatic pressure measurements, etc.) showed a chylous ascites related to portal hypertension, and left ventricular dysfunction was the only probable cause found.

**Conclusion:**

Asymptomatic heart dysfunction can mimic liver disease and should be kept in mind as a cause of chylous ascites.

## Background

Ascites is one of the most common complications of liver diseases, even though in 15% of the cases it is related to extrahepatic diseases; 3% are of cardiac nature and they appear associated with signs and symptoms of heart failure. It is also associated with certain blood laboratory abnormalities. In this cases, the ascitic fluid shows a high albumin gradient (>1.1 g/dl) and high concentrations of proteins [1-4].

We report an asymptomatic patient with chylous ascites as the only symptom of heart failure

## Case presentation

A 70 year old man was admitted with more than one year history of abdominal distension and a weight gain of 10 kilograms. He was evaluated in another center and was given the diagnosis of ascites associated with cirrhosis.

His past medical history was significant due to acute myocardial infarction in 1974, mild asthma and paroxysmal atrial fibrillation. Since his last evaluation, he has been receiving diltiazem 180 mg/day, aspirin 100 mg/day, digoxin 0,750 mg/week and spironalactone 50 mg/day. He is asymptomatic and walks 2000–3000 meters a day without angor or dyspnea.

The physical examination shows moderate abdominal distension, with no hepatosplenomegaly or edema, and there is mild jugular vein distension (1/3). The hepatojugular reflux was negative. His blood pressure is 130/70 mmHg, pulse 70/ min and regular. The cardiac auscultation is normal and his lungs are clear. His current weight is 78.8 kg. One year before this admission he weighted 70 kg and before starting diuretics he was weighting 81 kg.

The blood laboratory was normal, except for a slightly increase in gamma-glutamyltransferase and 5'nucleotidase (Table 1). A paracentesis was performed which showed a milky fluid with a high albumin gradient showing portal hypertension (Table 2), all cultures and cytological studies were negative. Other studies showed normal iron values; antiHIV, HBsAg, antiHBs, antiHBc IgM and antiHCV negative, antiHBc IgG positive; ANA, AMA, SMA y ANCA negative.

A helical CT scan of the abdomen showed mild hepatomegaly, mild enlargement of caudate lobe and ascites. An upper endoscopy ruled out esophageal varices and portal hypertensive gastropathy. A Doppler ultrasound showed a patent portal vein, with a normal diameter (6.6 mm) without alterations in the flow. A cardiac ultrasound showed moderate to severe systolic left ventricle dysfunction with global hypokinesia and the inferolateral wall was akinesic. The left ventricle diameters were normal and the left atria was enlarged (52 mm). The estimated left ventricular ejection fraction was 35%. There were no relevant valve signs, nor mitral or tricuspid regurgitation, nor pericardial disease.

A liver biopsy showed engrossment of centrilobular veins walls and fibrosis, with centrilobular hemorrhage and marked sinusoidal dilatation in acinar zone 3, suggesting increased intrahepatic venous pressure. An angiogram showed dilated but patent suprahepatic and portal veins and inferior vena cava, with slow flow. The measure of free suprahepatic, wedge suprahepatic, pulmonary capillary wedge, pulmonary artery, right ventricle and right atrial pressures confirmed the diagnosis of portal hypertension related to heart failure (Table 3). He began treatment with furosemide 40 mg/day and spironalactone 100 mg/day; he continued treatment with aspirin, digoxin and diltiazem. The patient lost 8 kg and the abdominal distension was resolved approximately 2 month after the beginning of the treatment. Given the good response to treatment we decided to withhold other possible treatments (i.e. angiotensin-converting enzyme inhibitors).

## Discussion

Chylous ascites is an unusual type of ascites (<1%) featured by the presence of high concentration of triglycerides in the ascitic fluid (>200 mg/dl). The most common causes in adults are disseminated neoplasia and lymphomas, traumatic or surgical rupture of lymphatic vessels, and less frequently it is associated with cirrhosis. In this last case, its pathogenic mechanism is unknown, but it could be related to degenerative changes related to age (it is most frequently seen in elderly patients) and hypertension in lymphatic vessels caused by portal hypertension [1,5,6].

Liver involvement in chronic heart failure is common and it will depend on the type and severity of cardiac disease. In patients with moderate to severe heart failure, 95% show hepatomegaly, 75% peripheral edema, 20–25% pleural effusion and up to 25% show ascites. Ascitic fluid has a high albumin gradient with a high concentration of proteins, usually more than 3 g/dl. Blood laboratory shows mild increases in ALT and AST (5%), mild elevations in bilirubin (20–80%), high alkaline phosphatase (10–20%), prolonged prothrombin time and decrease in albumin levels (30–50%) [7,8].

These disorders appear with a symptomatic cardiac disease which, in general, was previously diagnosed. Even tough there are cases of heart diseases presenting as liver diseases [9-11], this is the first case of chylous ascites caused by heart failure, with no signs or symptoms of cardiac disorder. There are some cases associated with constrictive pericarditis [12-14] and with severe heart failure [15,16]; all these patients showed signs or symptoms manifesting cardiac involvement (pulmonary edema, jugular vein distension, etc.). There are two mechanisms probably involved: an increase in the abdominal lymph production and an ineffective development of collateral flow. High venous pressure increases the abdominal lymph production due to an augmented capillary filtration and, even tough lymphatic flow increases in response, the augmented central venous pressure reduces lymphatic drainage. Unlike mechanical obstruction of the thoracic duct, where the development of lymphaticovenous collaterals channels provides lymphatic drainage, generalized central venous hypertension caused by cardiac disease prevents the development of an effective collateral flow. Given that heart failure is a common disorder and chylous ascites is a very unusual one, other unknown mechanisms should participate in its pathogenesis.

In this case, other probable causes were ruled out: the long evolution (more than a year) and various images studies ruled out the presence of a hidden neoplasia; there is no recent traumatic or surgical history; the angiogram ruled out vascular liver disease and liver biopsy didn't show any specific liver disease or cirrhosis.

Taking into account all the performed diagnostic procedures and the good response to diuretic treatment, left ventricular dysfunction is the main probable cause. Historically, chylous ascites caused by neoplasia or lymphoma, was associated with poor prognosis and was difficult to treat. Other causes of chylous ascites should be kept in mind, because the prognosis and the response to treatment depend on the disease that causes it.

## Conclusion

Chylous ascites is an uncommon type of ascites and has various causes. Asymptomatic heart dysfunction should be kept in mind as a possible cause of chylous ascites and, also, as a cause of liver disease of unknown etiology.

## Competing interests

The author(s) declare that they have no competing interests.

## Authors' contributions

ER and OGM have made substantial contributions to acquisition of data, analysis and interpretation of data; have been involved in drafting the manuscript or revising it critically for important intellectual content; and have given final approval of the version to be published. Each author has participated sufficiently in the work to take public responsibility for appropriate portions of the content. All authors read and approved the final manuscript.

## Pre-publication history

The pre-publication history for this paper can be accessed here:



## Figures and Tables

**Table 1:** Laboratory values.

Variable	On admission (reference value)
Hematocrit (%)	49.5
Hemoglobin (g/dl)	15.7
Erythrocyte sedimentation rate (mm/h)	2 (3–10)
White cell count (per mm^3^)	9800 (4000–10000)
Platelet count (per mm^3^)	219000 (150000–400000)
Prothrombin time (%)	99 (70–100)
Bilirubin total (mg/dl)	0.94 (<1.2)
Bilirubin direct (mg/dl)	0.26 (<0.36)
AST (UI/L)	12 (<23)
ALT (UI/L)	13 (<25)
Alkaline phosphatase (UI/L)	159 (<207)
Gamma-glutamyltransferase (UI/L)	89 (<38)
5'nuceotidase (UI/L)	20 (<9)
Total proteins (g/dl)	7.8 (6.6–8.7)
Albumin (g/dl)	4.7(3.5–5)
LDH (UI/L)	191 (160–320)
Creatinine (mg/dl)	1.17 (<1.2)
Urea (mg/dl)	39 (<50)
Cholesterol total (mg/dl)	200 (<220)
Triglycerides (mg/dl)	135 (<190)

**Table 2:** Laboratory values in ascitic fluid.

Variable	
Aspect	Milky, turbid
Proteins (g/dl)	5.6
Albumin (g/dl)	3.4
Albumin gradient (g/dl)	1.3
Glucose (mg/dl)	123
LDH (UI/L)	115
Cholesterol (mg/dl)	141
Triglycerides (mg/dl)	814
Leukocyte count (per mm^3^)	3000
Neutrophil count	<10%

**Table 3:** Pressures (mm Hg) recorded during catheterisation

	Systolic	Diastolic	Media
Free suprahepatic	13	10	12
Wedge suprahepatic	16	13	15
Pulmonary capillary wedge	30	14	22
Pulmonary artery	43	17	27
Right ventricle	42	11	24
Right atrium	22	10	17
